# How much territory can a single *E. coli* cell control?

**DOI:** 10.3389/fmicb.2015.00309

**Published:** 2015-04-21

**Authors:** Ziad W. El-Hajj, Elaine B. Newman

**Affiliations:** Department of Biology, Concordia University, Montreal, QC, Canada

**Keywords:** *E. coli*, cell length, cell division, giant bacteria, metabolism

## Abstract

Bacteria have been traditionally classified in terms of size and shape and are best known for their very small size. *Escherichia coli* cells in particular are small rods, each 1–2 μ. However, the size varies with the medium, and faster growing cells are larger because they must have more ribosomes to make more protoplasm per unit time, and ribosomes take up space. Indeed, Maaløe’s experiments on how *E. coli* establishes its size began with shifts between rich and poor media. Recently much larger bacteria have been described, including *Epulopiscium fishelsoni* at 700 μm and *Thiomargarita namibiensis* at 750 μm. These are not only much longer than *E. coli* cells but also much wider, necessitating considerable intracellular organization. *Epulopiscium* cells for instance, at 80 μm wide, enclose a large enough volume of cytoplasm to present it with major transport problems. This review surveys *E. coli* cells much longer than those which grow in nature and in usual lab cultures. These include cells mutated in a single gene (*metK*) which are 2–4 × longer than their non-mutated parent. This *metK* mutant stops dividing when slowly starved of *S*-adenosylmethionine but continues to elongate to 50 μm and more. FtsZ mutants have been routinely isolated as long cells which form during growth at 42°C. The SOS response is a well-characterized regulatory network that is activated in response to DNA damage and also results in cell elongation. Our champion elongated *E. coli* is a *metK* strain with a further, as yet unidentified mutation, which reaches 750 μm with no internal divisions and no increase in width.

## Introduction

*Escherichia coli* has astonished investigators with its remarkable metabolic efficiency packed into such a small size. In its 0.5–2 μ length, it packs its genetic material, its metabolic machinery, and an impressive variety of adaptive strategies. It can make a new cell as fast as every 30 min with scarcely an error. The brilliant analysis of *E. coli* function by Jacob, Monod, and Lwoff excited the entire field and led to the amazing detail with which *E. coli* is now understood (Cohn, 2014).

In order to produce a new cell, *E. coli* must approximately double its cell contents and distribute them between 2 daughter cells. It must exactly duplicate and segregate its DNA, and it must double its length and divide itself at midcell. It becomes longer using a cell wall synthesizing system based on penicillin binding protein 2 (PBP2) to elongate. This elongation is the result of the combined activity of peptidoglycan synthesis and hydrolysis enzymes, which constantly remodel the cell wall, but the net result is an increase in cell length (Johnson et al., 2013). The direction of cell wall synthesis changes when the length has doubled, uses a different enzyme system based on PBP3, and coincides with synthesis of a septum at midcell. This system, known as binary fission, is thus an alternation between elongation via a PBP2 complex and division via a PBP3 complex (Lutkenhaus et al., 2012).

Initiation of the septum at midcell involves spatial inhibitors that prevent septum formation elsewhere, such as SlmA involved in nucleoid occlusion (Du and Lutkenhaus, 2014), and the well-known MinCD complex (Ghosal et al., 2014). Although the mechanisms by which they inhibit division are relatively well understood, how *E. coli* finds its mid-point in the first place has been a long standing problem. This was settled very recently by the lab of Suckjoon Jun, who showed that it divides when it has added a constant volume, the rate depending on how fast its environment allows it to do so (Taheri-Araghi et al., 2015). The cell alters its volume and length according to its environment. However in whatever conditions it can grow, it makes viable cells and wastes nothing, i.e., except for the end products of metabolism, it does not overproduce and excrete metabolic products.

## Various Ways to Grow Very Long *E. coli*

It would make sense that if one were to inhibit the activation of the PBP3 divisome in such a way that everything else functions, the cell would not divide, nor would it stop growing. It would continue its various metabolic functions and become very long. This is indeed what seems to occur in our strain MNR2 (see Extremely Elongated *E. coli* Cells). In this section we will discuss the problems that such an elongating cell might face, and assess the role of two amino acids (methionine and L-serine) and of alternative cell envelope components in promoting elongation.

### Potential Problems in Elongation

The elongating cell has a number of problems, and some might be expected to increase in severity the longer it gets. Among these, it has to add peptidoglycan to the wall, it has to synthesize and distribute DNA, it has to transcribe from the new and old DNA, make new ribosomes and distribute those, and use them to make new proteins and enzymes, and form new enzyme complexes and distribute them.

As the cell lengthens, its mass clearly must increase and each addition it makes to its protoplasm and its peptidoglycan must be pushing against more mass and more wall. If this caused it problems, one might expect it to grow more slowly than a cell which divides every time it doubles, and perhaps more important, to slow down as it becomes longer. This effect might be lessened by the saving in time and energy occasioned by not making the vertical cell wall.

However, a cell elongates by adding many short lengths of new peptidoglycan at many points along its surface, thus providing new space for the macromolecules it is making, and for the duplicating DNA to move into. Because the additions are many and short, rather than few and long, elongation may require less forces. If the sites are numerous enough, they may not even have to have a fixed pattern to maintain a linear form overall. Otherwise there must be some pattern to where PBP2 acts. In any case this forms an unobstructed tube within which cytoplasmic components can move.

Many physical chemists, and others, suggest that *E. coli* cytoplasm is extremely crowded, and this is often supposed to interfere with diffusion and distribution of cell contents. By one estimate, its cytoplasm contains 200 mg/ml protein, 11–18 mg/ml DNA and 75–120 mg/ml RNA occupying 20–40% of the cell volume and affecting transit though much more “excluded volume” (Hasnain et al., 2014). They suggest this implies that it is not easy for even small molecules to make their way through the cytoplasm, and even harder for large molecules.

The effects of crowding are considered in interesting detail in a 1999 review (Hoppert and Mayer, 1999). These authors suggest that bacterial cells have functional compartments but they are not bounded by membranes. Instead they are formed from the nucleoid, from multienzyme complexes, from storage granules, and cytoskeletal elements all of which affect the properties of water in the cell (Hoppert and Mayer, 1999).

These papers, and many others, make it clear that the cell is crowded with respect to macromolecules. However, the rapidity of elongation suggests that this apparent crowding does not actually slow synthesis much or inhibit function of newly synthesized and distributed material, no doubt because of the continuous elongation of the tube. This is perhaps another example of *E. coli* being able to solve its problems better than we can understand them.

Indeed one of the problems of “crowded cytoplasm” considerations is that it does not allow for the nature of growing cells and turbulence within expanding cytoplasm. While the wall is being extended by hundreds of insertions, the DNA is being synthesized and segregated, and causing turbulence in the cytoplasm around it, which must aid the motion of other molecules in the cytoplasm. Small molecules can probably move with very little obstruction.

### Involvement of Methionine

We have found several cases in which production of long cells in *E. coli* is associated with alterations in methionine and *S*-adenosylmethionine (SAM) metabolism. The first long *E. coli* isolated in the Newman lab was derived from a supposed *metK* mutant described in R. C. Greene’s lab (Greene et al., 1973). This carried a point mutation in *metK* together with an *lrp* mutation which decreased Lrp expression and increased *metK* expression just enough to permit growth (Newman et al., 1998). We showed that a strain carrying only the *metK* mutation required leucine for growth, as an inducer of the *lrp* gene. When grown overnight with limiting leucine, e.g., 5 μg/ml, the cells did not divide but elongated up to 100-fold with imperfectly segregated nuclei and no visible constriction (Newman et al., 1998). Whereas other cell division mutants (such as strain MNR2 or *ftsZ84* mutants) made no septum at all, the SAM-starved cells incorporated three proteins into the septum, FtsZ, ZipA, and FtsA, but continued to elongate with these partial septa (Wang et al., 2005).

These cultures probably did not represent the full potential for elongation of the *metK* strain. The cells could elongate only once they reduced the leucine sufficiently to inhibit division, but then quickly ran out of leucine for protein synthesis. The 100-fold elongation is thus quite prodigious. We conclude that a supply of SAM is needed for complete activation of septum formation, and suggest that this is due to the need for one or more methyltransferase reactions in activating division.

We could not make a strain carrying a deletion of *metK* because *E. coli* cannot transport SAM, but we did construct a deletion compensated by a plasmid-carried inducible *metK* (Wei and Newman, 2002). However, the group of D. O. Wood created a SAM-transporting *E. coli* strain by isolating a SAM transporter from *Rickettsia prowazekii* (Tucker et al., 2003) and cloning it into an *E. coli* plasmid, allowing a mutant deficient in *metK* to grow in rich medium with an exogenous SAM supply (Driskell et al., 2005). They kindly allowed us to use this transport gene for all our work on SAM utilization including strain MNR2.

Using the *metK* deletion strain of Wei Yuhong, and the Wood plasmid, with some modifications, we were able to determine the growth requirements of an *E. coli metK* mutant (El-Hajj et al., 2013). The mutant in fact had two requirements, SAM because of the deletion, and methionine because in the presence of SAM, methionine synthesis is inhibited.

As we discovered with considerable difficulty and chagrin, using externally provided SAM is a matter of real complexity. We have described these problems in DETAIL (El-Hajj et al., 2013) and anyone wishing to use commercial SAM should read that description. We used commercial SAM, purified of course, for our experiments (before understanding all these complexities) but would make our own enzymatically for any further experiments.

After a year on the lab shelf, commercial SAM is a 1:1 mixture of its enantiomers *(R)*-SAM and *(S)*-SAM, even if it was provided as pure *(S)*-SAM. Enzymatic SAM is purely *(S)*-SAM when first made but isomerizes relatively quickly. Only *(S)*-SAM is a methyl donor for most *E. coli* methyltransferases. However, *(R)*-SAM provided exogenously is a methyl donor for one reaction, the methylation of homocysteine to methionine by the enzyme MmuM, a third methionine synthase.

We found then that the SAM deletion strain requires both SAM and methionine, but the amount of methionine it requires depends on the proportion of SAM which is in the form of *(R)*-SAM and that proportion varies greatly. However with more *(R)*-SAM than it needs, the cell still needs methionine though very little—about 100 ng/ml rather than 20–40 μg usually provided to a methionine-requiring mutant such as a *metB*. We do not understand what the cell needs the small amount of methionine for, when it is getting so much methionine from *(R)*-SAM and is not actually blocked in methionine biosynthesis.

We conclude that methionine metabolism is less well understood than generally thought, and that *(R)*-SAM can serve *E. coli* as a methyl donor for conversion of homocysteine to methionine via the enzyme MmuM. We suggest further that the switch from PBP2 elongation to PBP3 division requires the availability of methyl donors.

### Involvement of L-Serine

Among the oddities of L-serine metabolism is that *E. coli* codes for three different very specific high K_m_ L-serine deaminases (L-SDs) but cannot use L-serine as carbon source. These enzymes, SdaA, SdaB, and TdcG (Su and Newman, 1991; Shao and Newman, 1993; Burman et al., 2004), use a [4Fe-4S] cluster to catalyze the deamination of serine to pyruvate (Cicchillo et al., 2004). They are so highly specific for L-serine that even TdcG, which is coded by a gene in an anaerobic threonine utilization operon, does not deaminate threonine but is a dedicated L-SD (Burman et al., 2004). However, they only work on high concentrations of L-serine, preventing interference with peptidoglycan synthesis (Zhang et al., 2010).

Deleting all three genes from *E. coli* of course greatly increased the availability of L-serine and it also severely impeded the ability of the mutant to grow in minimal medium with amino acids, where the deletion mutant made very large, abnormally shaped cells (Zhang and Newman, 2008). In Luria broth, the triple mutant made long filaments—not as long as strain MNR2 but nonetheless an average of 10 × normal length and up to 80 ×. Most surprising, even though SAM normally cannot enter the *E. coli* cell, it prevents filament formation in the triple mutant equipped with the Rickettsial transporter (Zhang and Newman, 2008).

We suggest that high serine interferes with the serine/glycine/C1 balance of the cell and decreases the availability of C1 units and SAM for the cell division specific methylation resulting in production of long cells. On the other hand, overproduction of L-SD also produced long cells. The mutant with the highest serine deaminase activity recorded, the *ssd* mutant now shown to be a subclass of *cpxA* mutants (Rainwater and Silverman, 1990; Monette, 2006), grows in glucose minimal medium expressing high L-SD, with 50% of its cells as small rods and the other 50% as a few enormously long rods. This suggests that every now and then the cell cannot divide and does not recover from this but instead goes on elongating.

Neither of these cases is well understood. However, we suggest that when the balance of serine, glycine and C1 is not maintained, some methylation associated with cell division does not occur.

### Interference with Cell Division by Provision of Alternative Cell Wall Components

If the cell is provided with the means to make an entirely different cell wall, e.g., not peptidoglycan, it may go ahead and do it, and then find that it has problems breaking the alternative wall down into smaller cells. Like so many of us, Lederer et al. (2011) used *E. coli* to clone and express proteins of interest to them in *E. coli*. In this case, they cloned S-layer proteins from *Lysinibacillus sphaericus* JG-A12 into *E. coli* BL21(DE3). These are mostly protein and glycoprotein, forming the S-layer envelope of various organisms. *E. coli* can express them, grow well and produce viable cultures. However, its morphology is entirely changed (Lederer et al., 2011).

In early exponential phase these cells form filaments 100 μm long or more. Later these appear as transparent filaments with small *E. coli* rods lined up inside them. For the moment, there is not enough information to analyze these in detail. Nonetheless it is obvious that adding alternative cell wall components to the cell’s possibilities has interfered with peptidoglycan synthesis and allowed it to construct an alternative cell envelope of considerable length.

## Extremely Elongated *E. coli* Cells

### Isolation of a Mutant that Does Not Activate PBP3

*Escherichia coli* divides every 0.5 h or so in Luria broth with or without added sodium chloride (LB, LBNoSalt), and about every 58 min in minimal medium with glucose, all at 37°C. In our attempts to understand the requirement for methionine in the *metK* deletion mutant (see Involvement of Methionine), we have recently isolated a mutant which divides every 80 min in minimal medium but divides rarely in LB and almost not at all in LBNS. That is, it can elongate via PBP2 and divide with PBP3 when growing in minimal medium, but in LB or LBNS it can only use PBP2 and thus continues to elongate indefinitely. The longest cell we have seen so far was is 750 μ long (El-Hajj and Newman, 2015, Figure 1).

**FIGURE 1:**
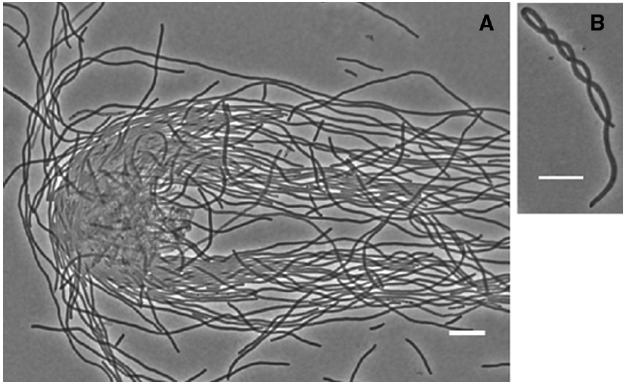
**Flexibility of the MNR2 eel cells. (A)** Intertwined eel cells of strain MNR2 incubated in liquid LBNS for 16 h. **(B)** Example of a corkscrew-shaped filament from a 24 h LBNS slide culture. Scale bars are 10 μm.

When the mutant, known as MNR2, is grown in minimal medium it makes the rods one expects, but they are considerably longer than the parent cell. When the population is subcultured into LBNS, every cell continues to elongate and cannot divide, so that they gradually grow longer over the next 15–24 h. This elongation occurs at many points along the cell (Woldringh et al., 1987). The exact pattern of elongation points is not known (den Blaauwen, 2013). However, even our longest cells show no distortion, so that points of elongation must be chosen according to some pattern. A biophysical simulation by Sean Sun and colleagues suggests that MreB stabilizes the peptidoglycan tube, and provides a framework for the localization of the PBP2 complex and its function (Jiang et al., 2011).

We attributed the elongation in MNR2 to the much lower osmotic pressure of LB and LBNS (El-Hajj and Newman, 2015). This was based on the fact that adding the components of LB to minimal medium produced a medium which had a high osmolality (349 vs. 342 mOSm/kg) and did not prevent division. Also, cells were much longer in LBNS than in LB (93 vs. 256 mOsm/kg). This may indicate that the elongation rate is faster at low osmolality. However, it is also possible that the elongation rate does not change but the probability of transient PBP3 activation is higher at the osmolality of LB.

The size of wild-type *E. coli* is larger the faster it grows (Chien et al., 2012). The larger size is required in order for the cell to contain all the machinery it needs to make cell components, among others, ribosomes to make protein at an increased rate. Thus when *E. coli* is taken from minimal to rich medium, it responds by growing wider and longer (Grover and Woldringh, 2001). Strain MNR2 of course grows longer, but the mechanism for this may be different since its width did not change in any of the thousands of cells we examined.

Cells subcultured in LBNS all elongate for the first few hours, with some extremely elongated (eel) cells reaching lengths up to 200 μm. These cells could be made to grow even longer by plating them on LBNS agar slide cultures, which provides them with fresh nutrients and is how we isolated the longest cell we have seen, 750 μm long. However, when plating these cells on the agar slides and examining them by time-lapse photography, a few produced a new rod at the end of the long cell (El-Hajj and Newman, 2015). These rods are about the size of the usual MNR2 rods in minimal medium, but they immediately begin to elongate again. This predilection for division near the ends is also seen in filaments formed in other ways (Dajkovic et al., 2010, movie S1 early frames). We conclude that PBP3 is normally inactive in the mutants in Luria media, and if it happens to function, it is quickly re-inactivated.

When we instead plated the eel cells on minimal medium slide cultures, we expected that PBP3 would immediately reactivate and they would start dividing promptly. Much to our surprise, the long cells continued elongating at multiple points along the cells, forming loops that pushed outward from the long axis of the cell (El-Hajj and Newman, 2015). This suggests that there are pre-requisites for PBP3 activation even when cells are over the length required to divide. How PBP3 function is activated is currently unknown. However, two separate studies have suggested that peptidoglycan synthesis is altered at the division point even before PBP3 activity begins. One group suggest that there may be PBP3 independent synthesis at midcell of a narrow band of new peptidoglycan, a process involving only two very early septation proteins, FtsZ and ZipA (Potluri et al., 2012). A second group shows that PBP2 and PBP3 interact directly for a short time, and suggests an association between them to synthesize a new area of peptidoglycan prior to the actual septation, so that both enzymes work concurrently for a short time as peptidoglycan synthesis shifts from PBP2 to PBP3 (van der Ploeg et al., 2013). The loops we have seen could be this new peptidoglycan being synthesized. In a small rod-shaped cell, this synthesis would simply push the poles apart and the cell would elongate in a straight line. However, the eel cells are much longer, and the adherence of the cell to the agar on the slide culture would require enough force to push away the entire length of the cell on either side, which could be up to 100 μm of cellular content. Peptidoglycan synthesis does not produce sufficient force to do this, so instead the force pushes in a direction perpendicular to the long axis of the cell and the area where it occurs loops outward.

The cell wall of *E. coli* is usually considered to protect the cell against osmotic pressure by virtue of its rigidity, and this applies to the usual small rods. The peptidoglycan at the poles (which is always the result of a former PBP3-driven lateral peptidoglycan synthesis during division) is thought to be inert. That is, old peptidoglycan is not degraded and replaced by newly-synthesized peptidoglycan (Koch and Woldringh, 1994). This is in contrast to the longitudinal peptidoglycan synthesized by PBP2, which undergoes constant recycling as older peptidoglycan is degraded by and newer peptidoglycan is incorporated in its place (reviewed in Johnson et al., 2013). In a small *E. coli cell* 1 or 2 μ long, the rigidity of the inert polar peptidoglycan maintains the characteristic unbending rod shape. However in the much longer MNR2 eel cells, the rigid poles are frequently separated by over 100 μm and as a result the cells are remarkably flexible. In fact we found cells can be intertwined even by the forces of pipetting a drop of a diluted culture onto a slide! This can be seen in Figure 1A, where the free ends of cells are aligned by the flow caused by the falling cover slip. The flexibility can also be seen in a single cell twisting around itself into corkscrew shapes (Figure 1B). Long chains of small *Bacillus subtilis* cells form similar corkscrew macrofibers of astounding flexibility, and their elasticity generates enough torque and force to allow self-propulsion when their natural unwinding is hindered (Mendelson et al., 2000). Neil Mendelson recorded a remarkable video of these bacterial macrofibers on his website (www.neilhmendelson.com), which unfortunately seems unavailable at the time of writing.

Whether the high flexibility of the MNR2 eel cells makes it more difficult to produce a crosswall away from the poles, or whether the inability of the cells to form the crosswall in the first place is what lends them this remarkable flexibility, seems to be a chicken-or-egg question. What is clear is that the ends of the cell somehow alleviate this problem, as seen by the transient activation of PBP3 on LBNS slide cultures discussed earlier. At the pole, the cell can take advantage of the rigidity conferred by the end wall, and using that, it requires constriction at only one point one normal cell length away to form a rod of the usual *E. coli* size. This would also allow for the MinCD complex to shuttle within its usual length (Lutkenhaus et al., 2012) and help in accurate placement of the division site, as well as subsequent formation of a functional divisome.

### Characteristics of Long Cells

We did not attempt to find conditions which allowed the cells to grow particularly long. The 750 μ cell we photographed (El-Hajj and Newman, 2015) extended over three microscope fields, and was simply the longest we happened to find with our usual growth media. There is no reason to suppose that this is the longest possible, though physical problems may eventually make lengthening more difficult.

It is odd that the diameter of the cell does not increase on subculture into LB or LBNS. One of the earliest findings in studies of *E. coli* adaptation is that wild type *E. coli* transferred from minimal to rich medium, gets both longer and wider (Schaechter et al., 1958). Though there are many circumstances in which *E. coli* can get wider, reviewed in a fascinating review of bacterial shape (Young, 2010), this widening is not seen with this mutant when grown on enriched medium. Strains derived from the same parent do become very much wider when overexposed to L-serine (Zhang et al., 2010) so this does not seem to be simply a strain difference.

The long cells are remarkably flexible, as seen in the corkscrew pictured here (Figure 1B). They nonetheless occasionally lyse, as is particularly visible on solid medium. Lysis could be due to adherence to or to other cells pulling them in a different direction from the one in which they elongate. When they lyse, the entire contents pour out leaving an empty tube. In one of our photographs (El-Hajj and Newman, 2015, Figure 2B), the point of lysis fell at a point where the cell formed a closed loop on an agar surface, and one could see the cell contents within that loop. Clearly we could adapt this to collect the entire content of a single cell, perhaps by micromanipulation with a microsyringe.

**FIGURE 2:**
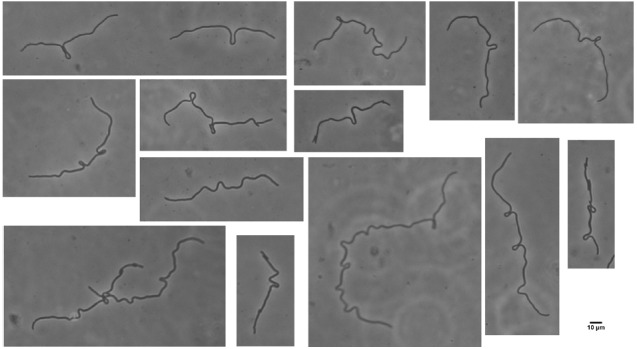
**Loop formation in the elongated cells after shift down.** MNR2 eel cells from a 16h LBNS culture were plated on minimal medium slide cultures and photographed after 2.5 h. Scale bar indicates 10 μm for the entire figure. Loops form at some distance from the end and from each other but otherwise show no obvious pattern.

We assume that as elongation goes on, new DNA is made and transcribed throughout the length of the cell (El-Hajj and Newman, 2015). As an example of “games” which become possible with cells of this size, it might be interesting to add an inducer like X-gal at different times and see if it can induce at all parts of the cell, or to streak inducer on the plate and let cells grow toward it. Similarly we plated cells on slides coated with turmeric powder and found that the cells appeared much thicker due to the negative staining, which would allow us to search for even longer cells at lower magnifications.

What is clear, and surprising, is how well these cells grow and metabolize even after many hours. They do not show signs of metabolic problems and the majority of the long cells can give rise to a colony (El-Hajj and Newman, 2015). This does not inform us as to how many of the DNA molecules in a long cell can give rise to colonies. It does tell us that the long cells remain alive and their metabolism lets them continue to elongate—i.e., their metabolic capacity is remarkable. As long as they are in LB or LBNS, we do not see any localized defect other than occasional lysis. As seen below, this is no longer the case when we transfer the cells to glucose minimal medium where they might be able to subdivide and multiply thereafter as rods.

### The Return of Elongated Cells to Minimal Medium

The elongated cells can reach the size of the largest bacteria known (see Giant Bacteria). As long as they are kept in enriched medium, the entire cell appears intact and functional. When they occasionally form rod-shaped cells at the ends of the long cells, these rods maintain the same characteristics, i.e., they elongate once again if left in rich medium. The remaining long cell also continues to elongate. There is no indication that there are non-functional areas in these long cells, with the possible exception that DNA does not segregate perfectly in all cells. However, even cells with smears of DNA elongate.

The situation is very different when the elongated cells are plated on minimal medium, where most of the cells give rise to a colony. However, this new colony is not derived from all parts of the elongated cells. Some parts do not grow at all, and some grow and die off, but each long cell has at least one area in which cells grow into a microcolony of rod shaped cells and ultimately into a colony with the same characteristics as MNR2.

We did not follow these in the detail they deserve but intend to do so. When plated on minimal plates, the cells did not immediately produce rods. As seen in Figure 2, the eel cell began to elongate, but by 2.5 h incubation, showed the problems we expected but did not see in rich medium. The new lengths of cells could not push the old cell mass to the sides but instead grew outward making irregularly spaced loops. These loops were randomly placed but the same process was seen in all dividing cells.

**FIGURE 3:**
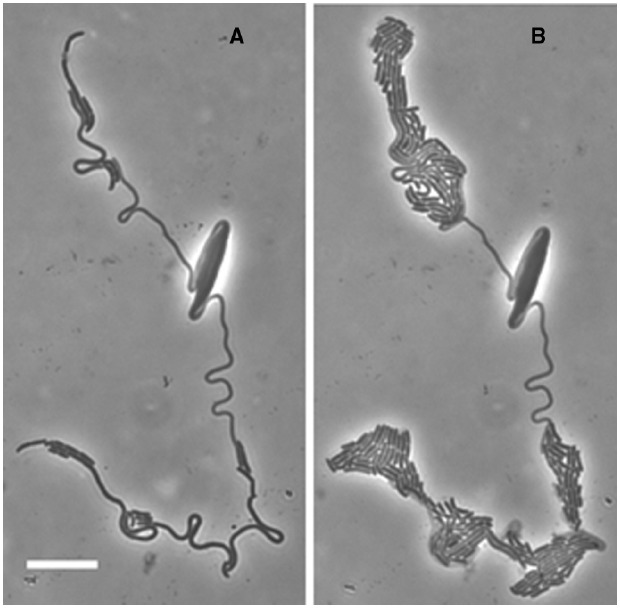
**Heterogeneity in the MNR2 elongated cells.** A 16 h culture of *E. coli* MNR2 in LBNS plated on a glucose minimal medium slide culture at 37°C was photographed after 6.5 h **(A)**, then left at room temperature for 18 h and the same field was re-photographed **(B)**.

We have not found other mentions of these loops in *E. coli*. This is the first evidence that the long cells are not entirely homogeneous, and this becomes obvious by 4.5 h when the cells produce a few rods, and break up into segments of various lengths, and some even lyse. An example of this extreme heterogeneity is seen in Figure 3. However, the design of this experiment actually promotes heterogeneity. This experiment is indeed a return to conditions which allow division, but it is also a major metabolic shift down. While the cells can suddenly begin to activate PBP3 and divide, they are also suddenly deprived of nutrients and have to begin making all the compounds they need. They can do this at a few points, but not at many. This experiment is worth repeating with a shift from LBNS to LBmin, i.e., restoring high osmolality in the presence of all the nutrients in LB.

## Filament Formation During Cell Division Arrest

Although the MNR2 eel cells we saw at low osmolality are the longest *E. coli* observed, conditional elongation in *E. coli* had been observed during the 1960s and 1970s, when it was characterized in mutants that were impaired in cell division. These mutants would continue elongating for a short time after division was blocked, but unlike the eel cells they would ultimately lyse and die.

Cell division requires the assembly of a complex division machinery. The process is initiated when FtsZ, the most well studied of the division proteins, polymerizes into a discrete structure, called the FtsZ ring or Z-ring, but likely much more complex than currently thought (Fu et al., 2010). Assembly of the Z-ring is essential for the recruitment of subsequent members of the division complex, including PBP3 (Errington et al., 2003). In conditions under which cells cannot form the Z-ring, PBP3 is not activated and they cannot form a crosswall, but they do grow into relatively short-lived elongated cells called filaments.

Many factors can disrupt the elongation-division cycle of *E. coli* and lead to formation of filaments. These include alterations in the stability or availability of the essential division proteins, especially FtsZ. Mutations in *ftsZ* or depletion of the protein due to DNA damage and subsequent triggering of the SOS response are both well-studied conditions that cause filamentation (Jones and Holland, 1984; Addinall et al., 1996). These differ from MNR2 eel cells, which are also blocked in cell division and elongate under adverse environmental conditions (in this case, low osmolality), but are viable and become much longer.

### *E. coli* Filaments in *fts* Mutants

The *fts* (filamentation temperature sensitive) series of mutants were isolated by virtue of their inability to divide when grown on LBNS at 42°C and have allowed the elucidation of many of the steps in Z-ring formation (Lutkenhaus et al., 2012). Such temperature-sensitive (Ts) mutations have been isolated in many of the essential cell division genes, which gave rise to the nomenclature of *fts* for these genes. Most of these mutants share common traits when shifted to 42°C, in that they begin cell division, and reach different stages depending on the mutation and the gene affected, but none of them can complete the process (Taschner et al., 1988). Because FtsZ is the earliest division protein, *ftsZ* mutants abort division very early on, which typically results in so-called “smooth” filaments. Disrupting the later division proteins, such as in *ftsA*, *ftsQ*, or *ftsE* mutants, allows the cells to begin septation. Most such mutants can polymerize FtsZ but cannot assemble the full complex required for successful division, and these cells assemble many partial, usually regularly-spaced Z-rings along the length of the filaments (Addinall et al., 1996). This results in “rough” filaments that show clear, marked constrictions where these incomplete rings form (Taschner et al., 1988, Figure 3). Regardless of what stage is inhibited, the filaments remain viable for only a few hours. The *ftsZ84* mutant, one of the earliest and best studied, lyses and sees a drop in viability 3 h after the temperature shift (Ricard and Hirota, 1973).

Much work was done with *ftsZ84* over the decades, taking advantage of the conditional mutation to study and understand FtsZ function and the essential role the protein plays in cell division (Lutkenhaus et al., 2012). Although most of these studies focused on FtsZ itself and its molecular role in cell division, they did in the process reveal a lot of information about how *E. coli* functions (or does not) when it reaches much longer lengths than usual. Most of the *ftsZ*(Ts) mutants can function at permissive temperatures such as 30°C. Under these conditions, the *ftsZ84* mutant synthesizes DNA and segregates nucleoids regularly (Ma and Margolin, 1999). The cells grow as rods and divide normally, although a significant number of cells (about one-third) lack Z-rings entirely (Addinall and Lutkenhaus, 1996). Upon shifting to 42°C, regular nucleoid segregation continues, but now occurs over the entire length of the filament (Bi and Lutkenhaus, 1992; Ma and Margolin, 1999). However, cell division is blocked with remarkable speed. FtsZ rings in an *ftsZ84* mutant seem to disappear as early as 2 min after the temperature shift, even though some of these cells retain a sharply-demarcated septum when viewed by electron microscopy (Addinall et al., 1997, Figure 2). After 10 min, these are replaced by deeper, blunted constrictions, which never complete the process. This is consistent with division sites beginning to form, but due to the mutated FtsZ the cells cannot complete the process at 42°C and septation aborts prematurely, leaving these blunted constrictions to mark the site of their failure.

As *ftsZ84* filaments are left at 42°C, they continue elongating and show no signs of rings (Addinall et al., 1996). If left for several hours they eventually lyse and die (Ricard and Hirota, 1973), but this fate can be avoided by shifting the filaments back to a permissive temperature (such as 30°C) before enough damage has been done. When cells of an *ftsZ84* mutant are upshifted to 42°C for 2 min, then downshifted back to 30°C, they start forming new rings at unconstricted, future division sites, but they seem unable to reform a ring at a previously-constricted site that was aborted during the upshift (Addinall et al., 1997). The cell “remembers” that it chose a division site and started to constrict, but it either cannot remember that the constriction aborted prematurely, or it cannot promptly resume or restart the constriction, presumably because the topology of an aborted site is different and a partial septum is already there and prevents reassembly of the Z-ring complex. Unfortunately no study followed these filaments for a longer time after the downshift, and whether the cells are eventually capable of fully recovering and re-targeting a previous division site if given enough time remains unknown. The MNR2 eel cells, with their increased viability and lengths, could provide a useful tool to settle this question.

*In vitro* studies of the mutated FtsZ84 protein have revealed defects in its biochemical function even at 30°C, but these do not seem to affect its ability to support cell division under permissive conditions *in vivo*. FtsZ is a GTPase and a tubulin homolog (De Boer et al., 1992; RayChaudhuri and Park, 1992). Like most members of this superfamily, it polymerizes by binding GTP and immediately hydrolyzing it, then depolymerizes when GDP is released (Scheffers and Driessen, 2002). The polymer structure is very dynamic and the turnover of FtsZ is high as individual molecules are cycled in and out of the polymer (Sun and Margolin, 1998). FtsZ84 has greatly reduced GTPase activity as seen by biochemical assays, even at 30°C, yet complete Z-rings are readily seen in *ftsZ84* mutants and the cells can divide (Mukherjee et al., 2001). Likewise, the Z-ring of an *ftsZ84* mutant has a much slower turnover rate than wild-type, but does not affect its doubling time at 30°C (Stricker et al., 2002). The biochemical defects of the mutant protein, which are measurable under all conditions, only manifest in the cell itself under specific environmental conditions. Unfortunately the exact players that compensate for these defects at 30°C yet allow the formation of the elongated filaments at 42°C remain unknown.

However, a couple of factors that can affect filamentation in *ftsZ84* have been well characterized. The first is that mild overexpression (twofold to threefold) of *ftsZ84* can suppress filamentation and allow *ftsZ84* mutants to divide at 42°C, which indicates that an increase in the level of the mutated protein can compensate for its functional deficit (Phoenix and Drapeau, 1988; Lu et al., 2001). The second is that many of the *fts*(Ts) mutations do not filament when grown in medium of higher osmolality. The *ftsZ84* mutant filaments in LBNS, but when grown on LB (i.e., with sodium chloride) and at 42°C, *ftsZ84* cells divide, are alive and form colonies (Ricard and Hirota, 1973). The inhibition of cell division in the MNR2 eel cells is similarly dependant on low osmolality, though unlike the *fts*(Ts) mutants they elongate without needing a temperature shift (El-Hajj and Newman, 2015). Not all the temperature-sensitive mutants are affected by osmolality or salt; for example *ftsZ26* filament at 42°C in both LB and LBNS (Bi and Lutkenhaus, 1992).

Why an increase in osmotic pressure restores division in some mutants but not others is not fully understood. In the MNR2 eel cells, elongation correlates with a decrease in the cellular levels of FtsZ, which we have suggested is the reason these cells cannot form a functional Z-ring (El-Hajj and Newman, 2015), but no such correlation has been drawn in any of the *fts*(Ts) mutants that we know of. On the contrary, in a number of *ftsZ*(Ts) mutants (including *ftsZ84*) grown in LBNS, the levels of FtsZ were *higher* at 42°C, when the cells filament, than they were at 30°C where these mutants could divide as the usual short rods (Addinall et al., 2005). *E. coli* grows within a large range of osmotic pressure but functions optimally between 300 to 500 mOsm (Cayley and Record, 2004). A change in osmotic pressure affects the cell at just about every level, from the composition of the lipid bilayer to protein-nucleic acid interactions and most enzymatic reactions (Kornblatt and Kornblatt, 2002). Low osmolality affects MNR2 and *ftsZ84* similarly, in that they both elongate, but the precise mechanism disrupted by this change in osmotic pressure is different and the cells become either filaments destined to die or eel cells that can keep elongating.

### Filament Formation During the SOS Response

As we mentioned in the introduction to this review, one of the remarkable features of *E. coli* (as well as many other bacteria) is that its metabolism is very efficient. New cells are produced rapidly with no wasting of metabolites and with very few mutants. The SOS response is a general stress response that many bacteria undergo when exposed to environmental or chemical factors that lead to DNA damage, such as radiation or some antibiotics (Walker, 1996). It involves a global and comprehensive regulatory network aimed at temporarily halting replication and division while repairing the DNA lesions, which ensures that the mutations that led to triggering the response do not get passed on to daughter cells.

The mechanism underlying induction of the SOS response is well described (Erill et al., 2007). Most of the genes involved in triggering the response are regulated by the LexA repressor, which is normally bound to their promoter region and blocks their expression (d’Ari, 1985). Conditions that induce DNA damage (such as UV irradiation) lead to the formation of lesions in the DNA. During DNA replication, the DNA polymerase complex inevitably encounters these lesions and cannot get past them, which stalls the replication fork. This leads to the recruitment of RecA to the site of the lesion and its activation when it binds to the single-strand DNA generated by the interruption in replication (Sassanfar and Roberts, 1990). Activated RecA can promote the autolytic cleave of LexA (Horii et al., 1981), which dissociates it from the promoter region of the SOS genes and enables their active transcription. A host of genes are then expressed (Janion, 2008), many of which are involved in stabilizing the stalled replication fork or in repairing the lesion (Baharoglu and Mazel, 2014). In order to buy time for repairing the DNA damage, the cell delays septation through the SOS-induced cell division inhibitor SulA (Huisman et al., 1984).

SulA blocks cell division during the SOS response by interacting directly with FtsZ in a 1:1 (Higashitani et al., 1995). This forms a stable complex that prevents FtsZ subunits from polymerizing and effectively inhibits septation at its earliest stage (Trusca et al., 1998; Cordell et al., 2003). SulA inhibition of FtsZ polymerization is reversible (Maguin et al., 1986), which allows the cell to exit the SOS phase and resume normal division once the DNA damage has been dealt with. This is controlled by the Lon protease, which plays a role in the degradation of SulA, freeing the FtsZ monomers to polymerize and initiate a new division cycle (Schoemaker et al., 1984).

If Lon function is inhibited, SulA is no longer degraded and can continue to accumulate and to sequester FtsZ. This extends the division block of the SOS response, and as the cells continue to elongate they form aseptate, multinucleate filaments (Adler and Hardigree, 1965). These filaments are not viable and the cell division block eventually becomes irreversible; within 4 or 5 h of their formation, only 2% of the filaments can recover and form colonies (Adler and Hardigree, 1964). However, the block can be overcome by reactivating Lon in the filaments (Walker and Pardee, 1967), since SulA would then be degraded, freeing FtsZ to polymerize and reinitiate cell division (Schoemaker et al., 1984).

Early during their characterization, SOS-induced filaments were noted for being metabolically competent despite being inhibited in cell division. They continue to elongate, increase mass, and synthesize nucleic acids and proteins (Walker and Pardee, 1967). Despite triggering the SOS state in response to DNA damage, they replicate their chromosomes and distribute DNA continuously and regularly throughout their length (Adler and Hardigree, 1965). Why then do they lyse and die within a few hours, while the metabolically competent MNR2 eel cells survive and continue to elongate well past this time? The SOS response triggers the expression of diverse genes, many of which are not necessarily involved in cell division but in regulating other aspects of *E. coli* metabolism and physiology (Janion, 2008). This probably affects the filaments in ways that were not measured or observed but that disrupt their metabolism and eventually leads to their death. This would be advantageous from an evolutionary perspective—a filament where the SOS response stays induced for so long is one that cannot repair the DNA lesions that triggered it in the first place and its death ensures the mutations do not get propagated to daughter cells. One feature they still share with the MNR2 eel cells is that in both cases elongation is initiated by a decrease in the availability of FtsZ.

The cellular concentration of FtsZ is constant throughout the division cycle, and is set at a critical concentration below which division is hindered (Rueda et al., 2003; Weart and Levin, 2003). Division begins not by synthesizing more FtsZ but by assembling the heretofore soluble FtsZ into the Z-ring (Sun and Margolin, 1998), with eventual activation of PBP3. A decrease in FtsZ can prevent PBP3 activation and onset of division and lead to the formation of elongated cells, as seen when cells are partially or completely depleted of FtsZ (Dai and Lutkenhaus, 1991). In a typical *ftsZ*(Ts) mutant such as *ftsZ84*, the cells form filaments with no constrictions after transfer from 30° to 42°C (Addinall et al., 1996), where FtsZ84 function is inhibited. The filaments produce increased levels of FtsZ84, perhaps in an attempt to compensate for the inhibition in the activity of the mutant protein (Addinall et al., 2005). During the SOS response, the FtsZ molecule itself is intact, but its sequestration by SulA decreases the pool of available, active FtsZ, which also leads to filamentation (Lutkenhaus, 1983; Chen et al., 2012). A similar mechanism is used by OpgH, a moonlighting enzyme that acts as a nutrient-dependant regulator of cell size by sequestering FtsZ under nutrient-rich conditions (Hill et al., 2013). In the case of the MNR2 eel cells, FtsZ activity is reduced because the levels of the proteins are much lower in medium of low osmolality, presumably lower than the critical concentration required for division (El-Hajj and Newman, 2015). A reduction in FtsZ function is therefore clearly correlated with division inhibition and elongation, but the differences underlying the exact mechanisms in each case also lead to different fates for the elongated cells.

## Giant Bacteria

Although MNR2 grows as long as any bacterium when it is cultured in low-osmolality LBNS, it has no way of dividing in this medium. This distinguishes the *E. coli* long cells from naturally-occuring giant bacteria, which can divide despite reaching similar lengths. The giant bacteria have evolved complex mechanisms to survive and take advantage of this enormous size, which is indeed the norm for them.

When we think of bacteria, we tend to think of unicellular microorganisms 1–5 μm in length or diameter. Many of the “traditional” bacteria fall within this length, such as *E. coli*, *Salmonella*, *Vibrio, Bacillus*, and *Staphylococcus*. However, bacterial sizes encompass a much wider range. *Mycoplasma pneumoniae*, one of the smallest known bacteria, is only 0.2 μm in diameter. These cells are so small that, over the course of its evolution, the bacterium drastically reduced the size of its genome by loss of most of its metabolic pathways, locking it into an obligate parasitic lifestyle for the acquisition of most metabolites (Himmelreich et al., 1996). At the opposite end of the spectrum, *Epulopiscium fishelsoni* cells can reach lengths of 700 μm and widths of 80 μm (Angert et al., 1993). The largest bacterium known, *Thiomargarita namibiensis*, is a giant sulfur bacterium that forms linear chains of spherical-shaped cells with diameters of up to 750 μm, so large that individual cells can be seen without any optical enhancements (Schulz et al., 1999).

### *Thiomargarita* and Giant Sulfur Bacteria

Giant bacteria need to deal with very different surface/volume considerations and have evolved elaborate transport and division systems in order to survive at sizes much larger than the more typical bacteria. Koch attempted to estimate the maximum size a cell could be if it relied exclusively on diffusion, and calculated this maximum as 300 μm (Koch, 1996). The diameter of *T. namibiensis* cocci frequently exceeds this and can reach values as high as 750 μm. They overcomes transport problems by organizing their cytoplasm around a large storage vacuole that takes up almost 98% of the cell’s volume (Schulz et al., 1999). The actual cytoplasm is a thin spherical shell that wraps around this liquid vacuole and contains a large number of sulfur globules. This arrangement allows all points of the cytoplasm close proximity to both the extracellular space and the vacuole, which overcomes the diffusion size limit. Another giant sulfur bacterium was isolated in the Gulf of Mexico, with large spherical cells up to 375 μm in diameter. This *Thiomargarita*-like bacterium had a similar cellular structure consisting of a central storage vacuole surrounded by a spherical shell of cytoplasm, which also contained sulfur globules (Kalanetra et al., 2005).

Why do the *Thiomargarita* bacteria organize their cytoplasm in such a way, and why do they need such a large central vacuole? A very long cell with a small diameter, such as the MNR2 eel cells, can overcome surface/volume limitations while maintaining a much smaller biovolume. The central vacuole seems to be a feature of giant sulfur bacteria and had already been described in *Beggiatoa* (Nelson et al., 1989). *Beggiatoa* is motile and usually uses oxygen as an electron acceptor in its sulfur oxidation, but it can store nitrate in the vacuole and use it as a temporary alternative electron acceptor when oxygen is low and it needs to move to another, more oxygen-rich environment (Dunker et al., 2011). Both of the identified *Thiomargarita* bacteria are non-motile and their vacuoles are much larger than in *Beggiatoa*. They may have evolved these giant vacuoles as nitrate storage tanks to survive long periods of anoxia and of low environmental nitrate. Schulz and Jorgensen (2001) calculated that the vacuole of *T. namibiensis* could store enough nitrate for a cell to survive almost 2 months without an external supply of either oxygen or nitrate, but under laboratory conditions a cell can survive for well over a year.

### Epulopiscium fishelsoni

Before the identification of *Thiomargarita*, the previous contender for largest bacterium was *Epulopiscium fishelsoni*, a symbiotic microorganism that spends the entirety of its life cycle within the gut of the surgeonfish (Fishelson et al., 1985). Reaching a length up to 700 μm and a width of 80 μm, the cell encloses a large volume of continuous cytoplasm, resulting in transport problems from outside and within the cell (Angert et al., 1993). *Epulopiscium fishelsoni* overcomes surface/volume limitations using extreme polyploidy; each cell has between 50,000 and 120,000 copies of the chromosome, with the exact number being directly proportional to the size of the cell (Mendell et al., 2008). As *Epulopiscium fishelsoni* cells get larger they replicate their chromosome to such high numbers to allow extremely high expression of transport pumps, as well as ribosomes, many of which are concentrated toward the center of the cell, away from the cell membrane. This is coupled to a large number of infoldings of the cell membrane, which is thought to enhance transport across the membrane and further into the center of the cell (Clements and Bullivant, 1991). Transport pumps on the cell membrane are similar to those in other bacteria, but they are much more kinetically active and have unusually high transport rate (Bresler and Fishelson, 2006), presumably to compensate for *Epulopiscium fishelsoni*’s much lower surface/volume ratio.

*Epulopiscium* does not divide like the typical *E. coli* using FtsZ and PBP3 as we described earlier. Instead, two daughter cells form inside the parent cell, each with its own cell membrane, and both completely enclosed within the cytoplasm of the parent (Angert, 2012). FtsZ does polymerize into a ring structure, not at midcell, but at the poles of the daughter cells instead (Angert and Clements, 2004). Eventually the daughter cells take over most of the cytoplasm of the parent and burst out, killing the parent in the process (Montgomery and Pollak, 1988). The growth of these daughter cells inside the parental cell shares some similarities with endospore formation in bacteria such as *Bacillus* (Errington, 1993), with one major difference: *Epulopiscium fishelsoni* daughter cells are not quiescent but fully active. This makes *Epulopiscium* and its relatives unique in that they rely on viviparous reproduction with the formation of multiple internal offsprings, which are released by destroying the parental cell.

### Desulfobulbaceae and Cable Filaments

Recently, a relative of *Desulfobulbus* was identified that blurs the line of what defines an individual bacterial cell. These Desulfobulbaceae form extremely long cable-like filaments in marine sediments, up to 1.5 cm long, twice that of the longest MNR2 eel cell (Pfeffer et al., 2012). Although an individual eel cell has no compartmentalization and a continuous cytoplasm, the Desulfobulbaceae filament consists of a linear chain of hundreds of cells, each only 3 μm long and each with its own inner membrane. However, all the cells within the filament are encased within the same outer membrane, which spans the entire 1.5 cm of length uninterrupted. Whether each filament is a single cell or a colony of hundreds of cells depends on whether we define the inner membrane or the outer membrane as enclosing a single cell. The cytoplasm of each short cell within the filament is unconnected to that of the adjacent cells, but each cell is connected to its neighbors by a filling that bridges the gap (Pfeffer et al., 2012). This organization allows high electron conductivity, which gives the Desulfobulbaceae filament the remarkable property of acting like an electric cable capable of conducting current across the layers of sediment where it normally grows. When followed over several weeks, the filaments grow and expand deeper into the sediments (Larsen et al., 2014; Schauer et al., 2014). The remarkable length of these filaments is thought to help them couple oxygen consumption, which requires contact with surface sediments, and the sulfide oxidation that occurs in deeper, anoxic layers.

## Concluding Remarks

*Escherichia coli* normally exists as a small rod, but we have discussed many conditions that cause it to elongate when cell division is inhibited. Some of these are related to lowered activity of FtsZ, such as in *ftsZ*(Ts) mutants, or when FtsZ is sequestered by SulA during the SOS response. Many metabolic defects also interfere with cell division, including metabolism of amino acids such as serine and methionine, as well as disruptions SAM synthesis, which is required for most methylations. In the MNR2 eel cells, disruption of SAM metabolism linked to one or more unknown mutations has resulted in the longest *E. coli* cells observed. This might be due to the methylation of a key cell division activator being disrupted in this mutant.

We conclude from these and other examples that as long as it remains narrow, allowing efficient uptake and excretion of material, *E. coli* can arrange its metabolism to function and grow over large lengths of undivided protoplasm. It can place its DNA and ribosomes so as to produce mRNA and proteins, and organize its enzymes spatially to function efficiently. In these ways it is as efficient as the giant bacteria like *Thiomargarita*, which have evolved as very large cells and have specific mechanisms to live at these sizes. *E. coli* lacks the specific adaptation to divide but has no trouble surviving at such sizes.

What an ultra elongated *E. coli* cannot do that *Thiomargarita* can is divide! We suggest that long length presents no particular metabolic problems by itself. However, the lack of an efficient division system for its long variants keeps *E. coli* short.

### Conflict of Interest Statement

The authors declare that the research was conducted in the absence of any commercial or financial relationships that could be construed as a potential conflict of interest.
